# Spatio-temporal variation in nesting success of colonial waterbirds under the impact of a non-native invasive predator

**DOI:** 10.1007/s00442-018-4270-8

**Published:** 2018-10-13

**Authors:** Marcin Brzeziński, Piotr Chibowski, Joanna Gornia, Grzegorz Górecki, Andrzej Zalewski

**Affiliations:** 1 Faculty of Biology, University of Warsaw, Żwirki i Wigury 101, 02-089 Warsaw, Poland; 2 Mammal Research Institute, Polish Academy of Sciences, 17-230 Białowieża, Poland

**Keywords:** Great crested grebe, *Podiceps cristatus*, Breeding ecology, Coloniality, Predation, Invasive species, American mink, *Neovison vison*

## Abstract

Colonial breeding in birds provides protection from predators and may be particularly important when birds have to cope with an invasive predator. The probability of nest predation in a colony can vary depending on several factors, such as the nest’s location in the colony and the level of aggregation of nests. We studied the nesting success of colonial great crested grebes and monitored the occurrence of the non-native invasive American mink in the colony. From among 92 grebe nests, 54.3% were successful. The daily survival rate (DSR) of grebe nests was positively affected by the increasing distance between the nest and lake shoreline, and negatively affected by the increasing distance between the nest and the five nearest grebe nests. The probability of mink occurrence in the colony increased with consecutive days of the breeding season and decreased with increasing distance from the lake shoreline. The DSR of grebe nests decreased with the increasing probability of mink occurrence along the shoreline distance gradient and the day of the breeding season. The results of the study confirm the impact of the American mink on waterbirds during the breeding season but also indicate that large breeding colonies are partially safe from mink predation, and that nest accessibility and the dilution effect influence the probability of nest survival. Our data suggest that the limited access to safe breeding sites on large lakes that can supply adult grebes and their chicks with food may affect bird productivity and population numbers at the landscape level.

**Electronic supplementary material:**

The online version of this article (10.1007/s00442-018-4270-8) contains supplementary material, which is available to authorized users.

## Introduction

Colonial breeding in birds and other animals is an evolutionary and ecological phenomenon and is always a trade-off between costs and benefits (Danchin and Wagner 1997; Sachs et al. 2007; Brown 2016). A variety of ecological conditions may favour its evolution, and among several factors that have been suggested to drive colonial breeding, a reduction of predator pressure is one of the most important (Kruuk 1964; Patterson 1965; Hoogland and Sherman 1976; Ims 1990a; Wiklund and Andersson 1994). However, it is not evident whether colonial breeding in birds always reduces predation: some studies have shown a negative relationship between nest predation risk, and nest aggregation or colony size, and others have found contrasting trends or no relation (Andersson and Wiklund 1978; Götmark and Andersson 1984; Hogstad 1995; Picman et al. 2002; Varela et al. 2007). Large colonies are often easy to detect by predators via auditory, olfactory and visual cues and may even attract predators (Rodgers 1987; Varela et al. 2007). The high detectability of colonies can be compensated by a reduction of nest predation deriving from group vigilance, communal defence (social mobbing) and the dilution effect (Götmark and Andersson 1984; Elliot 1985; Robinson 1985; Brown and Brown 1987; Ims 1990a; Murphy and Schauer 1996). Moreover, synchronal breeding may strengthen these defence mechanisms. In the absence of surplus killing, predators usually depredate only a small number of nests per visit and overall nest losses due to predation are low (Nisbet 1984), although, mass nest predation and brood consumption by large predators has been also reported (Drent and Prop 2008).

Social mobbing and the dilution effect may reduce predation, but just as important is the locating of breeding colonies in safe sites (e.g. on small islands far from shorelines) that prevent predator access to the colony. However, colonies are rarely completely inaccessible, and their isolation, which can be achieved by locating colony in a habitat hardly accessible to predators or by increased distance from shoreline, affects predator activity in the colony. Moreover, this activity may change over time according to the duration of the breeding season and availability of prey in a colony. If the probability of predator occurrence increases rapidly at the beginning of the breeding season (i.e. the occurrence probability curve has a concave shape), we can expect that a large number of nests will be lost due to predation. If the probability of predator occurrence increases slowly and accelerates over time (i.e. the occurrence probability curve has a convex shape), a larger number of nests will survive. Therefore, the nesting success of colonial waterbirds is related to the degree of colony isolation (it affects predator access), and the way in which predators respond to colonies (how fast they cue on prey).

Invasive predators usually impact breeding populations far more than native predators, and this phenomenon is explained by the naïve prey hypothesis (Salo et al. 2007). Novel predators have unpredictable effects on the benefits of coloniality, either taking advantage of prey aggregations, or at the other extreme, forcing prey to adopt coloniality to dilute predation risk. One of the most successful invasive carnivores introduced to Europe, Asia, and South America is the American mink *Neovison vison*. Mink predation on birds and their nests results in a decrease in breeding success and is considered the leading cause of decline of local populations of several waterbird species (Nordström et al. 2002, 2003; Banks et al. 2008; Peris et al. 2009; Niemczynowicz et al. 2017).

Behavioural changes in naïve prey seem to be a crucial strategy for reducing invasive species predation; however, such changes are only possible if a species displays behavioural flexibility that enables an advantageous response in confrontations with an invader (Zuk et al. 2014; Berthon 2015). In birds, a very important factor in reducing predation risk is plasticity in nest site-selection and breeding behaviour (Erwin et al. 2001; Forstmeier and Weiss 2004; Mainwaring et al. 2014), and the ability to switch from solitary to colonial breeding seems to be an effective form of this plasticity. Mink activity can trigger changes in the distribution of nesting sites and nesting behaviour of various waterbird species (Olsson 1974; Andersson 1992; Kilpi 1995; Landgren 1996; Craik 1997; Hario 2002; Barros et al. 2016).

In the Mazurian Lakeland, north-eastern Poland, mink established a wild-living population in the mid-1980s (Żurowski and Kammler 1987). In consequence, the impact of mink has led to a considerable decline in the coot *Fulica atra* breeding population in this region (Brzeziński et al. 2012). The breeding population of the great crested grebe *Podiceps cristatus* has not declined in number, but grebes have withdrawn from several small lakes and created new colonies or enlarged existing ones on a few large lakes. This species is a facultative colonial breeder, which means it can switch from solitary to colonial nesting, and predation pressure may be a factor that affects this choice of nesting strategy. In the pre-mink period, grebes in the Mazurian Lakeland nested both colonially and solitarily (Bukacińska et al. 1993), but the distribution of nesting grebes was more even and the proportion of solitary pairs within the breeding grebe population was higher than in recent years. By 2002/2003, 20 years after the mink invasion, the proportion of grebes breeding in colonies had increased, with about 90% of pairs nesting in colonies and 10% solitarily (Brzeziński et al. 2012). The switch in nesting behaviour was probably related to breeding success, as brood losses in birds breeding in colonies were 26% lower than those recorded in solitarily breeding grebes (Brzeziński et al. 2012). However, grebe colonies are not completely safe from mink predation; mink with home ranges overlapping grebe colonies depredate eggs and hunt chicks and adults (Brzeziński 2008).

To understand the factors shaping breeding success in grebe colonies we analysed spatio-temporal variation in mink activity in a colony in relation to the spatio-temporal variation in nest-survival rate. The aim of this study was threefold: (1) to estimate great crested grebe nesting success in a colony in relation to the duration of breeding season and spatial factors (aggregation, and distance from shoreline and colony centre), (2) to analyse the spatio-temporal shape (linear, convex or concave) of mink activity in the grebe colony over the breeding season, (3) to analyse the association between mink probability of occurrence in the colony and daily survival of grebe nests.

## Methods

### Nest monitoring

**Fig. 1:**
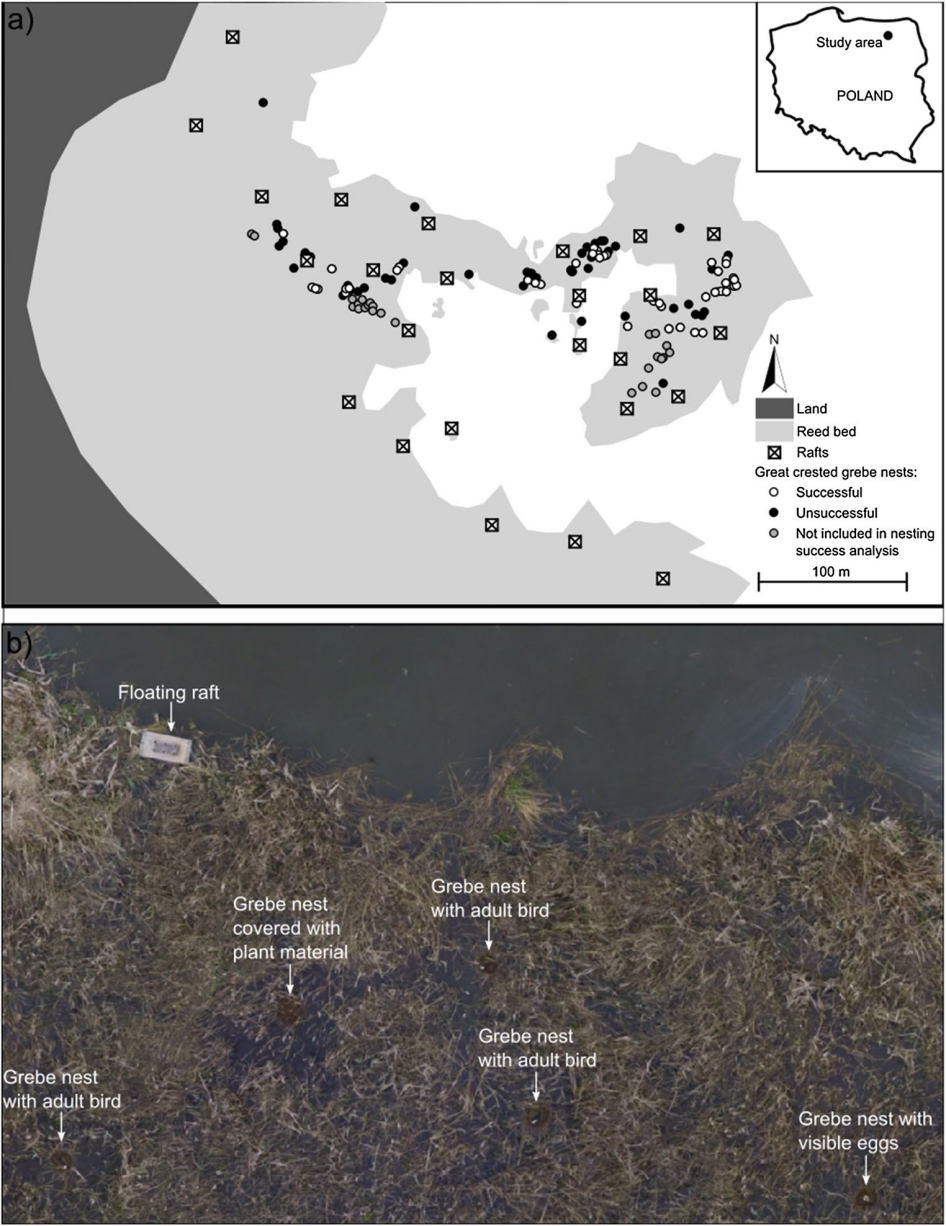
**a** Map of nest and raft distribution in the great crested grebe *Podiceps cristatus* colony at Lake Śniardwy, N–E Poland; **b** example of an aerial photograph of a small part of the study area with floating raft and grebe nests, made by a drone with a camera from an altitude of 10 m on the 15th May 2017. Nests that were not found in the beginning of the breeding season and were identified only after analysis of aerial photographs were not included in calculating DSR

The study was conducted in the Mazurian Lakeland (north-eastern Poland) at Lake Śniardwy, which is the largest lake in Poland (113.4 km^2^). The lake is eutrophic, and up to 23.4 m deep; its littoral zone is overgrown mostly by reeds *Phragmites australis*. The study area was located at the western bank of the lake, in Łuknajno Bay (53.78N, 21.62E) (Fig. 1a). The vast reedbeds, which are up to 400 m wide, reduce the impact of waves in the shallow bay, and create suitable nesting sites for waterbirds. The most common breeding species in the study area is the great crested grebe.

In 2017, great crested grebe nests were searched from 24th April until 17th June. Nests were assigned to the species on the basis of nest shape and the morphological features of eggs. The search was conducted by canoe. During the first check, nests were under construction and no nests with eggs were found. Nests were visited at 6–7 day intervals, and a total of nine visits were made to the colony until mid-June. The fate of each nest was recorded on successive visits until hatching or complete failure of the clutch occurred. A clutch was considered to be lost if all eggs were missing or damaged, or if the nest was abandoned or destroyed. We assumed that a nest had been predated when eggs disappeared without leaving remains, or when large pieces of crushed shells (especially with the presence of traces of yolk) were found in or around the nest; but the causes of clutch failure (i.e. predator species) were not determined. We treated nests as successful when at least one egg hatched.

The precise position of each nest was mapped using GPS. We then calculated nest parameters such as nest distance from the lake shoreline, nest distance to the geometric centre of the colony, and nest distance to the five nearest nests. Additionally, on the 15th May, a drone (Phantom 3 Advanced) with a camera attached was flown over the colony to take photographs of the whole study area from an altitude of 10 m (Fig. 1b). Aerial photographs were processed in Agisoft PhotoScan software and enabled the identification and counting of grebe nests not found in the beginning of the study, but which were used to calculate the aggregation index (see description below).

### Nesting success calculation

We estimated daily survival rates (DSR) for nests, using the nest survival module in the program MARK 6.0 (White and Burnham 1999) via the RMark package (Laake and Rexstad 2014) in R 3.4.2 (R Development Core Team 2014). This module uses a generalised linear model with a logit-link function and binomial errors to estimate daily nest survival probability with various combinations of covariates. We estimated model coefficients and log-likelihoods using a maximum-likelihood estimation. We tested the influence of nest distribution (distance from the lake shoreline, distance to the geometric centre of the colony and aggregation) on DSR. The level of aggregation was calculated for each nest as the mean distance to the five nearest nests. In this calculation we also included 25 nests that were identified from the aerial photographs. The DSR often varies during the incubation period in various ways (e.g. Bodey et al. 2011; Sexson and Farley 2012); therefore, we included daily survival as a linear or quadratic effect of the day in the breeding season. We included two-way interactions between variables (distance from the lake shoreline, aggregation, and day of breeding season). We did not consider more complex time trend models for fear of over-fitting the data (Dinsmore et al. 2002). However, we added two-way interaction between linear or quadratic effect of the day of the breeding season and the distance to the shoreline. This enabled us to test the hypothesis that over time, decreasing availability of food resources (grebe eggs) in area most accessible to mink, forces this predator to forage in the parts of colony more distant from the shoreline. As day 1 of the breeding season we used the earliest date on which a nest with an egg was found (2 May). We computed model support using Akaike’s information criterion with a correction for the small sample size (AIC_c_), and we evaluated the strength of the evidence for each model using normalised weights, *w*_*i*_ (Burnham and Anderson 2002). We selected the model with the smallest ∆AIC_c_ as the best among all compared models (50 models for DSR and five models for probability of mink occurrence); however, models within an ∆AIC_c_ of 2.00 were considered equally supported (Burnham and Anderson 2002). We reported results from two top models in Supplementary materials but acknowledging model uncertainty we calculated model-averaged estimates of DSR final model sets (Burnham and Anderson 2002). However, we have not calculated the averaged beta estimates, as it is not a useful approach in the case of our results (Gooch and White 2014). We estimated the relative importance of each variable in the set of models by summing the Akaike weights over all of the models in which the parameter of interest appears (Burnham and Anderson 2002).

We calculated nest-survival rate for the whole breeding season as the average daily nest-survival rate in the breeding season calculated from model-averaged estimates to the power of the length of the incubation period in days. We assumed the great crested grebe’s incubation period was 27 days (Goc 1986).

### Mink monitoring

Monitoring of American mink occurrence was carried out with 25 floating rafts distributed in and around the colony. Rafts are floating wooden platforms with a clay plate in the middle that records the footprints of animals, and are a very effective method of monitoring mink abundance (Reynolds et al. 2004). Rafts could only be accessed by canoe: they were placed opportunistically among the reeds and tethered to reed stems. They were deployed on the 24th April, left until the 17th June, and checked on the same days as the nests. Altogether there were eight raft-checks. Mink visiting the rafts left tracks on the wet clay, and the clay tracking medium was smoothed after each check; therefore, during each check we recorded all tracks accumulated over a week (results show percentage of rafts with mink tracks and let us to calculate probability of mink occurrence in each day of the breeding season). The distance between the two nearest rafts ranged from 29 to 64 m, and their distance from the lake shoreline ranged from 29 to 379 m (mean 221 m).

To estimate the factors affecting mink distribution we used a generalised linear model with a binomial distribution using day of breeding season and distance from the lake shoreline as covariates. We also included an interaction between these covariates.

One of our goals was to estimate the influence of spatio-temporal mink occurrence on nesting success. However, in the DSR model we could not include variables changing over the breeding season, such as mink occurrence. Therefore, we calculated the correlation between DSR in subsequences of breeding season days (5, 15, 25, 35, 45) for various distances from the lake shoreline (100, 200, 300, 400 m) and the probability of mink presence in relation to the day of the breeding season and distance from the lake shoreline. The estimates of both DSR and probability of mink presence were calculated from the averaged full models set.

## Results

**Fig. 2:**
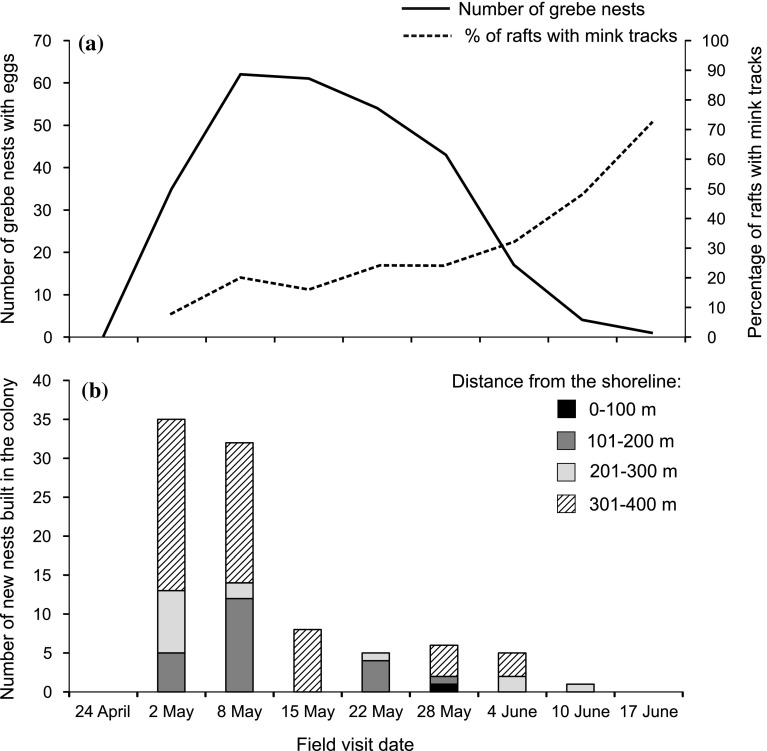
**a** Numbers of great crested grebe nests with eggs during consecutive nest-checks (days) in the colony at Lake Śniardwy and numbers of rafts visited by mink; **b** numbers of new nests built in the grebe colony

During the breeding season, we located 117 great crested grebe nests (by searching with a canoe and using aerial photographs) and 92 of them were monitored to estimate DSR. The shortest distance between a nest and the lake shoreline was 66 m, the furthest was 394 m, and the mean distance for all nests in the colony was 283 m. In the most clumped part of the colony the mean nest distance to the five nearest nests (level of aggregation) was 3 m. The mean distance between the most isolated grebe nest in the colony and its five nearest nests was 87 m, and the mean distance to the five nearest nests for the whole colony was 11 m. The aggregation of the nests did not correlate with distance from the lake shoreline; therefore, we used both variables in the models. The number of grebe nests with laid eggs in the colony increased over the first 2 weeks and then started to decrease (Fig. 2a). The majority of pairs built their nests between 300 and 400 m from the lake shoreline; however, most of nests at this distance range were built at the very beginning of the breeding season (Fig. 2b). Among all monitored nests, 50 were successful (54.3%) and 42 were unsuccessful (45.7%). Weather-related nest losses were not recorded.

**Fig. 3:**
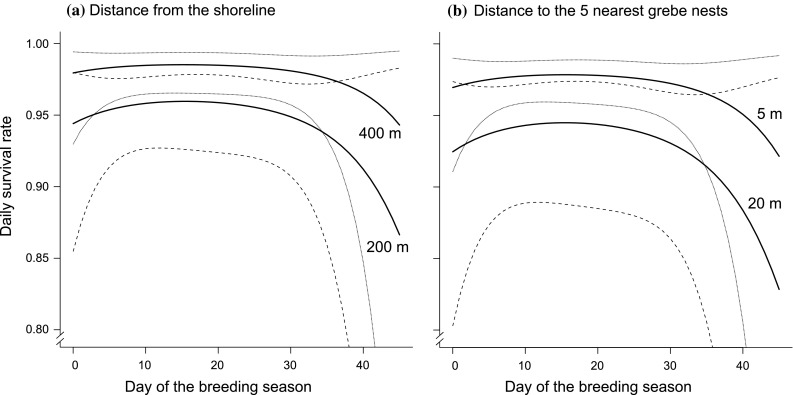
Daily survival rate estimated by the averaged models for nest survival plotted against the duration of the breeding season (in days) for two different values of the distance between the nest and lake shoreline (200 and 400 m) with a stable aggregation index (15 m) (**a**), and for two different values of the aggregation index (5 and 20 m) with a stable distance to the lake shoreline (200 m) (**b**). Dotted lines are the 95% confidence intervals. Day 1 = 2 May

Model selection provided support for seven models for grebe-nest survival rate (∆AIC_c_ < 2.00; Table 1, Supplementary materials, Table A). All competing top models included the distance from the shoreline and the distance to the five nearest grebe nests, five models included linear and four quadratic seasonal trends, two models included distance to colony centre (Table 1). One model included interaction between the distance from the shoreline and distance to the five nearest grebe nests, and one included interaction between the distance from the shoreline and quadratic seasonal trends. The DSR of grebes averaged across full set of models was stable over 30 days of the breeding season and then started to decrease over the following days (Fig. 3). It was positively affected by the increasing distance between the nest and the lake shoreline, and negatively affected by the increasing distance between the nest and the five nearest grebe nests (Fig. 3). The DSR increased from 0.9429 for the nests located 200 m from the shoreline to 0.9782 for the nests located 400 m from the shoreline, and decreased from 0.9687 for the nests with the average distance 5 m to the five nearest grebe nests to 0.9234 for the nests with the average distance 20 m. The relative importance of the variables calculated from the full set of models was 0.99 for the five nearest nests, 0.95 for the distance from the lake shoreline, 0.41 for the distance to the centre of the colony, and 0.49 for the quadratic and 0.19 for the linear seasonal trends. The average nest survival over the whole incubation period (27 days) calculated from DSR averaged across full set of models was 0.4421.

**Table 1:** Daily survival models of the great crested grebe nests at Lake Śniardwy

Model	*K*	AIC_c_	∆AIC_c_	*w*_*i*_	Deviance
Shore_dist + Nest_dist + *T* + *T*^2^	5	224.39	0.00	0.143	214.36
Shore_dist + Nest_dist	3	225.33	0.93	0.090	219.31
Shore_dist + Nest_dist + *T* + *T*^2^ + (Shore_dist × Nest_dist)	6	225.99	1.59	0.064	213.94
Shore_dist + Nest_dist + Colony_cent	4	226.01	1.62	0.064	217.99
Shore_dist + Colony_cent + Nest_dist + *T* + *T*^2^	6	226.11	1.71	0.061	214.05
Shore_dist + Nest_dist + *T* + *T*^2^ + (Shore_dist × *T*^2^)	6	226.27	1.88	0.056	214.22
Shore_dist + Nest_dist + *T*	4	226.38	1.99	0.053	218.36
Shore_dist + Nest_dist + *T* + *T*^2^ + (Shore_dist × *T*)	6	226.41	2.01	0.052	214.36
Shore_dist + Nest_dist + (Shore_dist × Nest_dist)	4	226.84	2.45	0.042	218.82
Shore_dist + Nest_dist + Colony_cent + (Shore_dist × Colony_cent)	5	227.17	2.78	0.036	217.13
Shore_dist + Colony_cent + Nest_dist + *T*	5	227.35	2.95	0.033	217.31
Shore_dist + Nest_dist + Colony_cent + (Colony_cent × Nest_dist)	5	227.38	2.99	0.032	217.35

Models are ranked by differences in Akaike’s Information Criterion for small sample size (∆AIC_c_) values. Covariates are as follows: *Shore_dist* distance from the lake shoreline, *Nest_dist* distance to the five nearest nests (aggregation index), *Colony_cent* distance to colony centre, *T* the day of the breeding season, *K* number of parameters, *w*_*i*_ Akaike weight. We only showed the top models, with ∆AIC_c_ < 3. See Supplementary materials for a full set of models

**Fig. 4:**
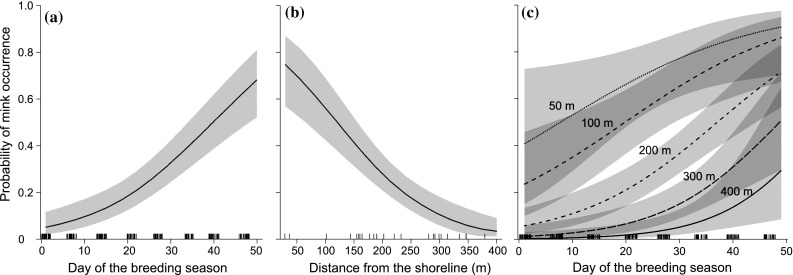
The probability of mink occurrence (with 95% CI) in relation to the day of the breeding season (**a**), and distance from the lake shoreline (**b**), predicted from the top generalized linear model; and the interaction between the day of the breeding season and distance from the lake shoreline (**c**), from the second most parsimonious model (Supplementary material Table B)

In 200 raft-checks (25 rafts checked 8 times), the presence of mink tracks was recorded 61 times, and mink tracks were not detected on 3 rafts (12%). Mink occurrence on the rafts slightly increased in May and steeply in June (Fig. 2a). Model selection provided support for two models of probability of mink occurrence. The top model included the day of the grebe breeding season and the distance from the lake shoreline (AIC_c_ = 186.91, *w*_*i*_ = 0.63). The probability of recording mink tracks increased with consecutive days of the breeding season, from 0.0478 (CI95% = 0.019–0.111) on the first day to 0.596 (CI95% = 0.455–0.722) on the 46th day, and decreased with increasing raft distance from the lake shoreline from 0.701 (CI95% = 0.530–0.829) for the rafts placed 50 m from the bank to 0.034 (CI95% = 0.013–0.090) for the rafts placed 400 m from the bank (Fig. 4a, b). The second competitive model was only 1.09 AIC_c_ units worse (AIC_c_ = 186.91, *w*_*i*_ = 0.37) than the top model and included an interaction between the day of the grebe breeding season and distance from the lake shoreline (Supplementary material Table B). At 50 m from the lake shoreline, the probability of mink occurrence in the colony was high in the first days of breeding season and increased over the following days; this increase had a concave shape (Fig. 4c). At 400 m from the lake shoreline the probability of mink occurrence increased slowly and the curve had a convex shape. According to model-averaging and the relative importance of the variables (Table 2), the probability of mink occurrences related to the day of the grebe breeding season and the distance from the lake shoreline. For two-way interaction the relative importance was 0.37 and confidence interval bounded zero.

**Table 2:** Model-averaged parameter estimates, standard errors (SE), and 2.5–97.5% confidence for each variable carried over into the final model set explaining the probability of mink occurrence in the great crested grebe colony

Parameter	Estimate	SE	2.5%	97.5%	*P*	Relative importance
Intercept	− 0.07725	0.78536	− 1.62235	1.45393	0.9145	
Shore_dist	− 0.01353	0.00417	− 0.02164	− 0.00535	0.0012	1.00
*T*	0.06501	0.02595	0.01456	0.11599	0.0117	1.00
*T* × Shore_dist	0.00015	0.00016	− 0.00016	0.00045	0.6523	0.37

*Shore_dist* distance between the raft and the lake shoreline, *T* the day of the breeding season

**Fig. 5:**
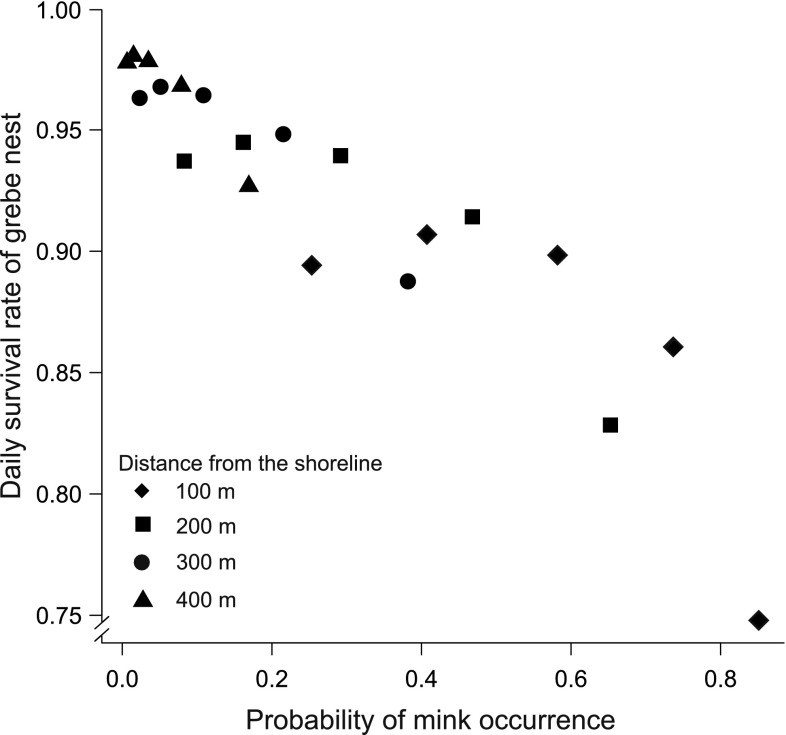
The relation between daily survival rate of grebe nests and the probability of occurrence of mink in consecutive days (5, 15, 25, 35, 45) of the breeding season and the distance from the lake bank (100, 200, 300, 400 m). The effect was calculated from the averaged models of daily survival rate and probability of mink occurrence

The analyses of DSR in relation to the probability of mink occurrence in subsequent days of the breeding season for various distances from the lake shoreline showed that the daily survival rate of grebe nests decreased with the increasing probability of mink occurrence (*R*^2^ = 0.843, *p* < 0.001; Fig. 5).

## Discussion

The results of the study showed that the DSR of great crested grebe nests depended on the nest’s location in the colony: it was higher for nests that were (1) located further from the lake shoreline and (2) more aggregated. During the breeding season, mink activity in the grebe colony increased with time, and the probability of mink occurrence in the colony was negatively related to the distance from the lake shoreline; thus the DSR was also negatively related to the probability of mink occurrence. The results of our study confirm the impact of mink on waterbirds during the breeding season but also indicate that large breeding colonies are partially safe from mink predation, and that nest accessibility and the dilution effect influence the probability of nest survival.

### Distance from the lake shoreline

Generally, colonial breeding by the great crested grebe reduces nest losses (Brzeziński et al. 2012); however, there is some evidence that the degree to which colonial breeding swamps predators and thus reduces predation may be overestimated, as bird colonies can be easily detected by many predators via auditory, olfactory, and visual cues (Rodgers 1987). We suspect that great crested grebe colonies are easily detected by mink, mainly due to the vocal activity of the grebes. Furthermore, nests of the great crested grebe are relatively large platforms and can probably easily be spotted by mink if they are in large aggregations, regardless of their location in the reeds. Nevertheless, grebes that located their nests further from the shoreline had higher nesting successes and our results are the first to show that the distance of the nest from the lake shoreline is an important factor affecting probability of nest survival in colonial waterbirds. The colony at Lake Śniardwy is in a shallow bay overgrown by extensive reedbeds that are up to over 400 m wide, and this creates a relatively safe place for breeding. However, in the Mazurian Lakeland, very wide reedbeds exist only on the largest lakes, which are not very numerous, so the sites suitable for colonial breeding and sheltering from mink predation are limited.

### Predator occurrence in the bird colony

Over the breeding season, the probability of mink occurrence in the grebe colony increased with time; therefore, we suspect that mink learn where food resources are available. However, at short distances from the lake shoreline this increase had a concave shape, causing an immediate decrease in DSR. At further distances from the lake shoreline (200–400 m), the probability of mink occurrence increased slowly (the curve had a convex shape); thus the predation pressure was relatively low until about the 30th day of the breeding season and increased only at the end of the season. The DSR of grebe nests was related to the probability of mink occurrence, and this suggests that nest losses in the colony mostly resulted from mink predation. The number of grebe nests with eggs increased in the colony over the first 10 days and after reaching a peak in mid-May started to decrease due to hatching and brood losses. It is likely, that as the number of nests with eggs decreased with time, mink were forced to look for them in areas more distant from the shoreline. However, the model including two-way interaction between linear effect of the day in the breeding season and the distance to the shoreline were 1.88 AIC_c_ unit worse than model without the interaction.

Mink is an inefficient swimmer and surface swimming is a very demanding process for this carnivore (Dunstone 1993); however it is able to swim relatively long distances (several hundred meters) (Niemimaa 1995; Salo et al. 2008). In the colony at Lake Śniarwy, mink access to the most distant nests requires long-distance and energy-consuming swimming, so the probability of mink occurrence in reedbeds decreases with distance from the bank because mink try to optimize the trade-off between hunting costs and food intake. Thus, grebe nests closer to the lake shoreline can be reached more easily by mink than those that are more distant and even if mink are aware of nests most distant from the shoreline (via auditory cues), they do not forage there until they can find nests that are easier to access.

Besides mink, there are two bird species that should be considered important nest predators in the grebe colony at Lake Śniardwy: the marsh harrier *Circus aeruginosus* and hooded crow *Corvus cornix*. It has been found that marsh harriers hunt grebes (Brzeziński and Żmihorski 2009), and hooded crows are known to take the eggs of numerous waterbird species, including great crested grebes (Salonen and Penttinen 1988; Zduniak 2006). Despite the fact that these two predators approach the colony from the land-side as do mink, they are avian predators, so the probability of nest predation by them is not related to the distance from the shoreline but rather to the density of vegetation cover, which protects nests from aerial detection. The avian predators’ impact increased variation in the data and thus the confidence interval of the nest DSR was relatively large (Fig. 3).

### Nest aggregation

Our results also showed that nest aggregation positively affected DSR. Generally, this finding is expected because in many colonial breeding birds, nesting success is positively related to the number of breeding neighbours (Murphy and Schauer 1996; Picman et al. 2002). Breeding neighbours reduce predator pressure, both by their occupation of nearby space (dilution effect) and, in the case of nest defending species, by their defensive and mobbing behaviours against predators. For the great crested grebe, the advantages of colonial nesting rather result from the high numbers and concentrations of nests, as predators can only find and depredate part of them. Nests in a colony are depredated by just one or a few mink individuals whose home ranges overlap the colony. Thus, large colonies may have lower overall mink predation.

The dilution effect increases nest survival and maximizes reproductive success when there is high synchrony of egg laying (Ims 1990a, b). Birds that breed synchronously with their neighbours generally have higher breeding success than those that breed earlier or later (Wittenberger and Hunt 1985; Murphy and Schauer 1996; Brunton 1999). In great crested grebe colonies the laying of the first egg by breeding pairs can be spread over a month; however, the majority of pairs lay their first eggs within about 2 weeks of each other (Konter 2005). In the great crested grebe, pairs that start nest building too early in the season may experience more brood losses than pairs that breed later (Konter 2008), and this rule was to some degree confirmed by our results. We found that the DSR was slightly higher for pairs that started to breed at the same time as the majority of birds, not very early and not very late. On the other hand, strong breeding synchrony may decrease food availability in the feeding grounds near the colony (Stempniewicz et al. 2000). To reduce intraspecific competition for food resources, a large colony can only be established on large lakes (such as Lake Śniardwy – 113.4 km^2^), where birds can scatter and find enough food for themselves and their hatched chicks.

### The central site in the colony

The selfish-herd hypothesis predicts that pairs breeding at the edge of a colony should suffer higher losses due to predation than pairs breeding in the centre because peripheral nests have less neighbours than central ones (Hamilton 1971; Hoogland and Sherman 1976). Central-periphery gradients of distribution, with high-quality pairs in the colony centre, are common in species breeding in homogeneous habitats that provide little variation in the physical quality of nest sites, but in heterogeneous habitats, central-periphery gradients can be disrupted and better quality sites can be located outside the centre of the colony (Minias 2014). In the grebe colony at Lake Śniardwy the best nesting patch was not in the colony centre but in the reedbeds most distant from the lake shoreline. Our results show that distance to the colony centre did not affect DSR. One explanation for this could be that the studied grebe colony was not very coherent. Therefore, the edge effect may be less important than other factors.

## Conclusions

The nesting success of great crested grebes breeding in colonies varies spatio-temporally. Because of the dilution effect and difficult access to nests, the impact of the American mink on grebes is reduced and the probability of nest survival in a colony depends on the nest’s location (it decreases with distance to the shoreline and increases with nest aggregation) and time of egg laying. Wide reedbeds in shallow bays seem to be relatively safe nesting sites; however, at most lakes the width of reedbeds is narrower than 100 m and many lakes are too small to supply large numbers of colonial breeding grebes with food. Therefore, suitable sites for colonial breeding, where overall nesting success is at least moderate, are very limited and this forces grebes to nest in large aggregations and may limit the breeding population at the landscape level. Although colonial breeding in grebes is an effective defence against mink, nesting in a colony may affect other aspects of their breeding ecology. Aggregation and synchrony in breeding season may increase aggressive interactions, food competition between birds or nest parasitism, and thus may affect overall reproduction output.

## Electronic supplementary material

Below is the link to the electronic supplementary material.

Supplementary material 1 (DOCX 22 kb)
