# Supplementary material for: Spatio-temporal variation in nesting success of colonial waterbirds under the impact of a non-native invasive predator
Source: Oecologia. 2018 Oct 13;188(4):1037–47. doi: 10.1007/s00442-018-4270-8 (PMC6244866; doi:10.1007/s00442-018-4270-8)
Supplement: Supplementary file 1 — Supplementary material 1 (DOCX 22 kb) [file 442_2018_4270_MOESM1_ESM.docx]

**Electronic Supplementary Materials**

**Spatio-temporal variation in nesting success of colonial waterbirds under the impact of a non-native invasive predator**

Marcin Brzeziński^1^, Piotr Chibowski^1^, Joanna Gornia^1^, Grzegorz Górecki^1^, Andrzej Zalewski^2^*

* corresponding author. Email: zalewski@ibs.bialowieza.pl

Table A. Summary of the model selection results of daily survival rate of great crested grebe nests. Models are ranked by differences in Akaike’s Information Criterion for small sample size (∆AIC_c_) values. Covariates are as follows: Shore_dist – distance from the lake shoreline; Nest_dist – distance to the 5 nearest nests (aggregation index), Colony_cent – distance to colony centre; T – the day of the breeding season. K – number of parameters; *w_i_* – Akaike weight.

| Model | K | AIC_c_ | ∆AIC_c_ | *w*_i_ | Deviance |
| --- | --- | --- | --- | --- | --- |
| Shore_dist + Nest_dist + T + T^2^ | 5 | 224.39 | 0.00 | 0.143 | 214.36 |
| Shore_dist + Nest_dist | 3 | 225.33 | 0.93 | 0.090 | 219.31 |
| Shore_dist + Nest_dist + T + T^2^ + (Shore_dist*Nest_dist) | 6 | 225.99 | 1.59 | 0.064 | 213.94 |
| Shore_dist + Nest_dist + Colony_cent | 4 | 226.01 | 1.62 | 0.064 | 217.99 |
| Shore_dist + Colony_cent + Nest_dist + T + T^2^ | 6 | 226.11 | 1.71 | 0.061 | 214.05 |
| Shore_dist + Nest_dist + T + T^2^ + (Shore_dist*T^2^) | 6 | 226.27 | 1.88 | 0.056 | 214.22 |
| Shore_dist + Nest_dist + T | 4 | 226.38 | 1.99 | 0.053 | 218.36 |
| Shore_dist + Nest_dist + T + T^2^ + (Shore_dist*T) | 6 | 226.41 | 2.01 | 0.052 | 214.36 |
| Shore_dist + Nest_dist + (Shore_dist*Nest_dist) | 4 | 226.84 | 2.45 | 0.042 | 218.82 |
| Shore_dist + Nest_dist + Colony_cent + (Shore_dist*Colony_cent) | 5 | 227.17 | 2.78 | 0.036 | 217.13 |
| Shore_dist + Colony_cent + Nest_dist + T | 5 | 227.35 | 2.95 | 0.033 | 217.31 |
| Shore_dist + Nest_dist + Colony_cent + (Colony_cent*Nest_dist) | 5 | 227.38 | 2.99 | 0.032 | 217.35 |
| Shore_dist + Nest_dist + Colony_cent + T + T^2^ + (Shore_dist*Colony_cent) | 7 | 227.42 | 3.02 | 0.032 | 213.35 |
| Shore_dist + Nest_dist + Colony_cent + (Shore_dist*Nest_dist) | 5 | 227.54 | 3.15 | 0.030 | 217.50 |
| Shore_dist + Nest_dist + Colony_cent + T + T^2^ + (Colony_cent*Nest_dist) | 7 | 227.67 | 3.28 | 0.028 | 213.60 |
| Shore_dist + Nest_dist + Colony_cent + T + T^2^ + (Shore_dist*Nest_dist) | 7 | 227.71 | 3.31 | 0.027 | 213.64 |
| Shore_dist + Nest_dist + T + (Shore_dist*Nest_dist) | 5 | 227.75 | 3.36 | 0.027 | 217.72 |
| Shore_dist + Nest_dist + T + (Shore_dist*T) | 5 | 228.34 | 3.95 | 0.020 | 218.31 |
| Shore_dist + Nest_dist + Colony_cent + T + (Shore_dist*Colony_cent) | 6 | 228.51 | 4.12 | 0.018 | 216.46 |
| Shore_dist + Nest_dist + Colony_cent + T + (Colony_cent*Nest_dist) | 6 | 228.71 | 4.32 | 0.017 | 216.66 |
| Shore_dist + Nest_dist + Colony_cent + T + (Shore_dist*Nest_dist) | 6 | 228.75 | 4.36 | 0.016 | 216.70 |
| Nest_dist + T + T^2^ | 4 | 229.19 | 4.80 | 0.013 | 221.16 |
| Nest_dist | 2 | 229.46 | 5.07 | 0.011 | 225.46 |
| Nest_dist + T | 3 | 230.91 | 6.52 | 0.006 | 224.90 |
| Nest_dist + Colony_cent + T + T^2^ | 5 | 231.03 | 6.63 | 0.005 | 220.99 |
| Shore_dist + T + T^2^ | 4 | 231.42 | 7.03 | 0.004 | 223.39 |
| Nest_dist + Colony_cent | 3 | 231.45 | 7.06 | 0.004 | 225.44 |
| Nest_dist + Colony_cent + T + T^2^ + (Colony_cent*Nest_dist) | 6 | 232.90 | 8.51 | 0.002 | 220.85 |
| Nest_dist + Colony_cent + T | 4 | 232.92 | 8.52 | 0.002 | 224.89 |
| Nest_dist + Colony_cent + (Colony_cent*Nest_dist) | 4 | 233.18 | 8.79 | 0.002 | 225.16 |
| Shore_dist + T + T^2^ + (Shore_dist*T) | 5 | 233.43 | 9.04 | 0.002 | 223.39 |
| Shore_dist + Colony_cent + T + T^2^ | 5 | 233.43 | 9.04 | 0.002 | 223.39 |
| Shore_dist + Colony_cent + T + T^2^ + (Shore_dist*Colony_cent) | 6 | 233.81 | 9.41 | 0.001 | 221.76 |
| Shore_dist | 2 | 233.99 | 9.59 | 0.001 | 229.98 |
| Shore_dist + T | 3 | 234.14 | 9.75 | 0.001 | 228.13 |
| Nest_dist + Colony_cent + T + (Colony_cent*Nest_dist) | 5 | 234.65 | 10.26 | 0.001 | 224.62 |
| Shore_dist + Colony_cent + (Shore_dist*Colony_cent) | 4 | 235.26 | 10.87 | 0.001 | 227.24 |
| Shore_dist + Colony_cent + T + T^2^ + (Shore_dist*T2) | 6 | 235.37 | 10.97 | 0.001 | 223.31 |
| Shore_dist + Colony_cent + T + T^2^ + (Shore_dist * T) | 6 | 235.44 | 11.05 | 0.001 | 223.39 |
| Shore_dist + Colony_cent | 3 | 235.51 | 11.11 | 0.001 | 229.49 |
| Shore_dist + Colony_cent + T + (Shore_dist * Colony_cent) | 5 | 235.83 | 11.44 | 0.000 | 225.80 |
| Shore_dist + Colony_cent + T | 4 | 235.87 | 11.48 | 0.000 | 227.85 |
| Shore_dist + T + (Shore_dist * T) | 4 | 236.12 | 11.72 | 0.000 | 228.09 |
| Shore_dist + Colony_cent + T + (Shore_dist * T) | 5 | 237.85 | 13.45 | 0.000 | 227.81 |
| T + T^2^ | 3 | 239.15 | 14.75 | 0.000 | 233.13 |
| Colony_cent + T + T^2^ | 4 | 239.92 | 15.53 | 0.000 | 231.90 |
| Intercept | 1 | 240.80 | 16.40 | 0.000 | 238.79 |
| T | 2 | 241.27 | 16.88 | 0.000 | 237.26 |
| Colony_cent | 2 | 242.58 | 18.19 | 0.000 | 238.58 |
| Colony_cent + T | 3 | 242.89 | 18.50 | 0.000 | 236.88 |

Table B. Beta estimates and standard error with confidence intervals from the two top models for the covariates of daily survival rate of grebe nests in the colony at Lake Śniardwy. For the covariates see Table 1.

| Variable | Estimate β | SE | LCL | UCL |
| --- | --- | --- | --- | --- |
| Top model (∆AIC_c_ = 0) | | | | |
| Intercept | 2.3096 | 0.7574 | 0.8251 | 3.7941 |
| Shore_dist | 0.0053 | 0.0020 | 0.0014 | 0.0092 |
| Nest_dist | -0.0589 | 0.0197 | -0.0975 | -0.0203 |
| T | 0.0962 | 0.0552 | -0.0121 | 0.2044 |
| T^2^ | -0.0027 | 0.0013 | -0.0052 | -0.0002 |
| Second model (∆AIC_c_ = 0.93) | | | | |
| Intercept | 3.0157 | 0.5772 | 1.8844 | 4.1470 |
| Shore_dist | 0.0049 | 0.0019 | 0.0011 | 0.0086 |
| Nest_dist | -0.0636 | 0.0192 | -0.1012 | -0.0260 |
